# Cross-lagged structural equation models for the relationship between health-related state and behaviours and body bullying in adolescence: findings from longitudinal study ELANA

**DOI:** 10.1371/journal.pone.0191253

**Published:** 2018-01-17

**Authors:** Viviane S. Straatmann, Ylva B. Almquist, Aldair J. Oliveira, Mikael Rostila, Claudia S. Lopes

**Affiliations:** 1 Department of Public Health and Policy,University of Liverpool, Liverpool,United Kingdom; 2 Department of Epidemiology,State University of Rio de Janeiro (UERJ), Rio de Janeiro, Brazil; 3 Center for Health and Equity Studies (CHESS), Stockholm, Sweden; 4 Laboratory of Social Dimensions Applied to Physical Activity and Sport (LABSAFE), UFRRJ, Seropédica, Brazil; Universita degli Studi di Catania, ITALY

## Abstract

We investigated the stability and the directionality of being body bullied and a set of four variables– 1) Body Mass Index (BMI), 2) moderate and vigorous physical activity (MVPA), 3) television time (TV) and 4) video game/computer time (VG)-, termed in the present study as ‘health-related state and behaviours (HRSB)’–across adolescence. The Adolescent Nutritional Assessment Longitudinal Study (ELANA) is a cohort study conducted among middle school students from two public and four private schools in Rio de Janeiro-Brazil. We analysed data from 2010 (T1) and 2012 (T2) among 810 adolescents (aged 9–15 at T1). Gender-specific structural equation models (SEM) were estimated, including autoregressive paths for the HRSB and being body bullied over time, correlations at T1 and T2, respectively, and cross-lagged effects. The results presented significant stability coefficients for almost all variables over time in both genders (except for MVPA in boys and girls and TV time among girls). There were positive correlations between BMI and being body bullied, as well as between TV and VG for boys (0.32, p<0.001 and 0.24, p<0.001, respectively) and girls (0.30, p<0.001 and 0.30, p<0.001, respectively) at T1. It remained significant at T2 (boys: 0.18, p<0.05 and 0.16, p<0.01; girls: 0.21, p<0.01 and 0.22, p<0.01, respectively). Examining the cross-lagged paths between being body bullied and HRSB, we observed that the reciprocal model provided the best fit for boys, indicating that BMI at T1 had a significant effect in being body bullied at T2 (0.12, p<0.05) and being body bullied at T1 had an effect on VG at T2 (0.14, p<0.01). Among girls the forward causation model showed the best fit, demonstrating a significant effect of being body bullied at T1 on VG at T2 (0.16, p<0.01). Apart from MVPA, both being body bullying and HRSB were largely stable across adolescence. For boys and girls alike, exposure to being body bullied seemed to increase their time spent on VG, while for boys BMI also predicted being body bullied. This study highlighted the complex interplay between being body bullied and HRSB and the importance of acknowledging gender differences in this context.

## Introduction

Bullying can be defined such as repeated physical, emotional, or verbal violent acts, which have hostile intent and involve an imbalance of power between aggressors and their victims [1]. There is a higher prevalence in less affluent countries, which have important inequalities in health, income and education. These socio inequalities seem to be a determinant of youth violence, able to influence the relationship between country wealth and bullying [2]. In Brazil, a wide middle-income country in Latin America, violence has assumed greater importance. While the magnitude and severity of violence rise in Brazil, in parallel, the interest in studies about bullying and teasing in youth grows [3, 4]. To address the vulnerabilities that impair the full development of this population, the School Health Program (*Programa Saúde na Escola*) was created in 2007 to contribute to the improvement of prevention, promotion, and health care actions [5]. Despite this, The National School of Health (*Pesquisa Nacional de Saúde Escolar—PeNSE*) revealed an increase in bullying in the Brazilian capital, from 5.4 to 6.8% between 2009 and 2012 [6].

Body bullying is one form of teasing about weight or shape particularly relevant for adolescents. Evidence suggest that being bullied because of weight or shape is reported by 19% of average-weight girls and 13% of average-weight boys, but much higher percentages are found in overweight girls (45%) and overweight boys (50%) [7, 8]. The peak period in which body bullying occurs has generally been recognised to be between ages 9 to 15 [9], with middle school consistently highlighted as the most prevalent time [10, 11]. Given the emphasis placed on weight and appearance in our society, this type of provocation may have serious psychological, physical, social, and academic implications [12, 13].

The Lancet Adolescents Health Series (2012) has presented a conceptual framework of adolescent health, emphasising the crucial importance of a life-course perspective in understanding adolescent’s health and development. This report highlighted the importance of the many health-related states and behaviours (HRSB) which usually start in the adolescence (tobacco and alcohol consumption, being overweight and obesity, physical inactivity and sedentary behaviours), and emphasised the complex interactions between these HRSB and social aspects involved in adolescent development [14].

Despite the fact that little is known about the relation between body bullying and HRSB in the context of middle-income countries, many scholars acknowledge that multi factors such as social, economic, and cultural [15] interplay, making the context much more complex [2, 14]. The bio-psychosocial model presented by Dodge and Pettit (2003) [16] reinforces this idea through the theory that reciprocal mechanisms between health outcomes and diverse factors (behaviours, biological dispositions, contexts and life experiences) could exacerbate or diminish antisocial development across time [16].

Regarding the pathways of being body bullied and HRSB, stigmatisation is more common among those in overweight or obese, often linked to inappropriate eating habits. It usually translates into pervasive victimisation and can increase vulnerability to a range of adverse outcomes as well as reinforce weight gain and impair weight loss efforts [12, 16]. Physical activity is another relevant HRSB in youths. Children who have been teased about their body tend to report a negative attitude towards physical exercise and, hence, reduce their physical activity levels [17]. The opposite mechanism can also occur when adolescents not engaged in an activity group or sports are intimidated, evince a potential reciprocal relationship [16]. While some bullied children avoid being engage in physical activities, others may appeal to excessive daily sedentary behaviours such as screen activities (e.g. television, video games, or computer use) as a way to escape of emotional pain, hide feelings [18]. Those that naturally prefer to spend time with quiet and lone activities can be bullied and may be rooted in much more rejection, anxiety and a difficulty of establishing relationships, instead of seeking activities that require relationships [19, 20]. In this context, it is important to emphasise that there are different types of screen time activities and it could widely vary by the socioeconomic and cultural factors of the country [21–23].

Gender is also a relevant aspect to be included in the research of body bullying during adolescence, since there are clear evidences of cognitive/ behavioural strategies and reactions differ between genders, especially from the beginning to the middle of adolescence. For example, girls report higher levels of social support (including instrumental and emotional support from others) and problem solving, whereas boys report higher efforts to disengage from the stressor (cognitive avoidance, avoidant action), e.g. ‘I tell myself it doesn’t matter’ [24]. As shown by Slater and Tiggemann (2011) [25], girls participate in organised sport at a lower rate than boys, as well as experience higher levels of teasing; both of them reported being teased by same-sex peers, but girls also reported being teased by opposite-sex peers (i.e. boys). Lastly, overweight girls and heavier children have been found to be exposed to more weight-related teasing than normal-weight boys [12, 26].

### Aims and research questions

Thus, the current study seeks to examine changes in being body bullied and four HRSB (BMI, MVPA, TV, and VG) across adolescence, and the directionality of these relationships by gender. Based on The Adolescent Nutritional Assessment Longitudinal Study (ELANA), this study used data of 810 students (aged 9–15 in 2010) collected at two time points (T1 = 2010 and T2 = 2012). These objectives resulted in the following set of research questions:

1. To what extent do a) being body bullied and b) HRSB remain stable from T1 to T2?
2. Are there associations between being body bullied and HRSB at T1 and/or T2?
3. Does being body bullied at T1 predict HRSB at T2 and/or do HRSB at T1 predict being body bullied at T2?
4. Are there significant gender differences in the models?

## Methods

This research was conducted according to the guidelines established in the Declaration of Helsinki and the Ethics Committee of Research of the Institute of Social Medicine of the State University of Rio de Janeiro (certificate number 0020.0.259.000–09) approved all procedures involving human subjects before the start of the examinations. Adolescents’ legal guardians provided a written informed consent. All the data we used were confidential and the researchers did not have access to any personal information that could identify individuals included in the dataset. Following the internal regulations of the ELANA Study committee, data is made available for specific research projects. Thus, we are not allowed to share the data we used for this study with other researchers. However, we are glad to answer any questions about the data used in this study and to share unpublished results.

### Study design and sample

The Adolescent Nutritional Assessment Longitudinal Study (ELANA) is a longitudinal study developed between 2010 and 2013 in 2 public and 4 private schools in the metropolitan area of Rio de Janeiro, Brazil. ELANA has two cohorts of adolescents: the middle and the high schools cohorts. The principal objective of the ELANA is to evaluate changes in anthropometric assessments and body composition, and identify relationships of factors such as health behaviours, socioeconomic and psychosocial conditions on nutritional status. This study included data from the middle school cohort at baseline in 2010 (T1) and third year of follow-up in 2012 (T2). At T1, a self-reported questionnaire was administered for the study of health-related states and behaviours (HRSB), body bullying, and socioeconomic variables among respondents. Body bullying and HRSB measurements were repeated at T2.

From the 946 adolescents initially identified in the enrolled schools, 888 met the eligibility criteria of the ELANA Study, which consider not having a physical or mental condition preventing the completion of questionnaires and/or not being pregnant or lactating at the time of data collection. Between the 888 eligible individuals, 32 (3.6%) refused to participate, 46 (5.2%) did not have parental consent, and 4 (0.45%) had no birth date information. The effective study sample comprised 810 students, which had sociodemographic and anthropometric information (main interest variables of ELANA Study) at baseline. From these 810 individuals, 797 adolescents completed information about body bullying at T1, corresponding to a response rate of 88.5%. At T2, 536 of the adolescents repeated the body-bullying questionnaire, indicating 33% missing cases.

We used imputation process of the maximum likelihood estimation to deal with missing cases in our interest variables. Thus, we carried out the statistical analyses with 810 adolescents at both times of the study. The maximum likelihood estimation is probably the most pragmatic missing data estimation approach for structural equation modelling. It has shown evidences of unbiased parameter estimates and standard errors for data missing at random and data missing completely at random (Enders, 2001). School changes by students are very common in Brazil, which turned difficult to follow the adolescents over time. It would suggest that the missing cases were probably not directly relate to the variables in investigation.

### Measures

#### Health- related state and behaviours (HRSB)

We assessed Body Mass Index (BMI (kg/m^2^)) through the direct measurements of weight and height [27]. The adolescents were barefoot and wearing light clothes. Weight was measured with an electronic scale to the nearest 0.1 kg (Kratos®, Embu das Artes, São Paulo, Brazil). Height was measured with a portable stadiometer, to the nearest 0.1 cm (Alturexata®, Belo Horizonte, Minas Gerais, Brazil) with the subject standing fully erect with feet together, head in the Frankfort plane, and with arms hanging freely during the measurement.

We assessed Physical Activity (PA) by the self-reported short version of the International Physical Activity Questionnaire–IPAQ. The short version of the IPAQ questionnaire is composed by eight open questions which estimates the weekly time spent in different intensities of PA in the last 7 days (e.g. “During the last 7 days, on how many days did you do vigorous physical activities?”; “How much time did you usually spend doing vigorous physical activities on one of those day’s activities?”). We multiplied the number of day for minutes spent in moderate and vigorous PA, separately. Then, we summed values of moderate and vigorous PA to access in an only variable minutes / day of moderate and vigorous physical activity (MVPA). Guedes et al. (2005) validated this instrument for Brazilian adolescents aged higher than 14 years [28]. Unusual and blank answerers (e.g. ten hours of MVPA per day) were checked by ELANA team and asked again to the students to avoid wrong classifications and lose of data. We performed the analyses with the variable of time (in minutes) of MVPA per day based on the guidelines for data processing and analyses of the IPAQ [29].

The television (TV) and video game/computer use (VG) times were obtained by a self-reported questionnaire, asking two questions: ‘How many days do you watch TV and use VG per week?’ and ‘In general, how many hours do you usually spend watching TV and using VG per day?’. For the first question, answers were categorised on a five-point scale for TV and VG: (1) almost never/ never; (2) 1 to 2 times per week; (3) 3 to 4 times per week; (4) 5 to 6 times per week; and (5) every day. We then transformed this into days per week: 1 = 0 days (almost never or never), 2 = 1.5 days, 3 = 3.5 days, 4 = 5.5 days, 5 = 7 days. We calculated the average of minutes per day of TV and VG variables, using the formula [(days per week)*(hours per day)]*60/7 [30].

#### Body bullying

We assessed body bullying by the Child-Adolescent Teasing Scale (CATS). The CATS was designed to measure self-perceived teasing and was intended to be used both as an outcome measure in research and as a screening tool in academic or clinical settings [31]. The domain of bullying about the body is composed of two items (‘*I am teased about my weight…*’ and ‘*I am teased about my body shape*…’) with two separate responses, one for frequency (‘*How much*: *…*’) and one for perceived harmfulness (‘*It annoys me*: *…*’), with four response options ranging from never (1) to a whole lot (4). The total score for each item ranged from 1 to 16 and is calculated by multiplying the frequency score by the corresponding perceived harmfulness score. Scoring was done in this manner because the frequency and perceived harmfulness components of teasing are synergistic and not additive relative to the amount of distress that can result from them. Children with higher body bullying scores perceive more teasing than children with lower scores.

### Covariates

We included socioeconomic and psychological well-being variables in the models, which were identified in the literature as potential confounders in the relationship between being body bullied and HRSB. Data of socioeconomic condition (type of school and asset ownership) and psychological well-being were obtained through a self-administrated questionnaire at T1. In Brazil, socio-demographic and economic characteristics are directly related to the type of school; for this reason, we utilised type of school (private or public) such as an indicator of socioeconomic condition. The economic characteristics of the family assessed by the Criterion of Economic Classification Brazil—Brazilian Association of Research Companies were also used thought an indicator constructed from information of presence or absence of on ownership of durable assets at home (TV; VCR or DVD player; radio; bathroom; automobile; washing machine; refrigerator; and freezer (independent appliance or part of duplex refrigerator). The weight attributed to the presence of each item is given by the complement of the relative frequency for each item; the rarer the presence of the item, the greater its weight [32].

Psychological domain of the KIDSCREEN-27 questionnaire was used to assess the psychological well-being of children and adolescents [33]. The questionnaire uses the following criteria to investigate psychological well-being: positive emotions, life satisfaction, and feelings of sadness or loneliness. The questionnaire poses questions concerning the past week and gives five options for answers on each item. The raw scores were used to calculate T values, based on the Rasch model [34].

### Data analyses

Firstly, the distribution of all variables of the sample was tested by the Kolmogorov Smirnov test. Due to the non-normal distribution of the sample, an asymptotic estimation (more appropriate for non-parametric distributions, without missing value imputation) was tested instead of the maximum likelihood missing values estimation. All the coefficient values remained similar in this analysis but lost their statistical significance, most likely due to the decreased sample size. Hence, it was decided to continue the analysis with the maximum likelihood for missing values estimation. The maximum likelihood estimation was used to deal with missing values, which is probably the most pragmatic missing data estimation approach for structural equation modelling, and has been shown to produce unbiased parameter estimates and standard errors for data missing at random and data missing completely at random. The process works by estimating a likelihood function for each individual based on the variables that are present so that all the available data are used. Model fit information was derived from a summation of values across fit functions for individual cases, and, thus, the model fit information is based on all cases. The maximum likelihood estimation estimates two models: the ‘unrestricted’ model, meaning that all variables are correlated, and the specified model. The difference between the two log-likelihoods is used to derive the chi-square. This approach allows one to use all the available information in the variables [35].

Comparisons between genders were performed through the non-parametric Wilcoxon Test, and Wilcoxon Rank Compared Test to evaluate differences between means ranks over time from T1 to T2. Meanwhile, before the more easily interpreted descriptive statistics (means, standard deviations, minimum and maximum) were presented, they were properly tested with adequate parametric tests, getting the same statistical results (p values) as from non-parametric tests (details available upon request).

**Fig 1:**
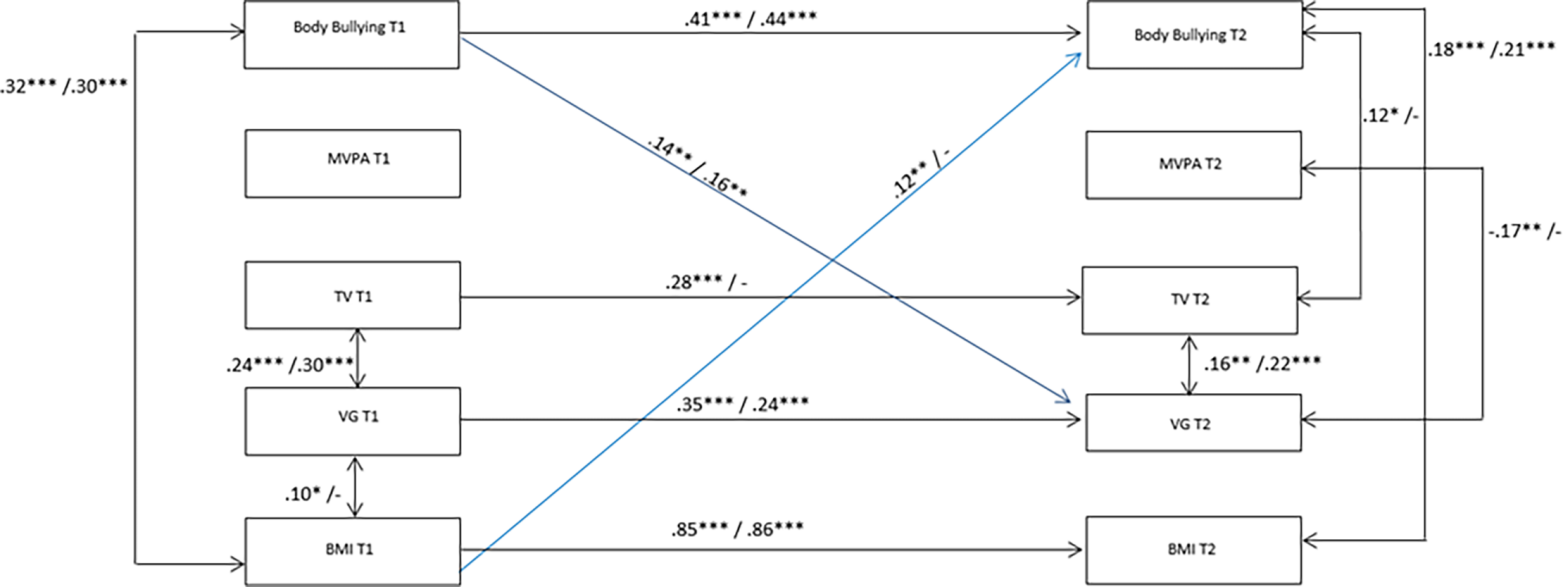
Baseline, forward causation, reversed causation and reciprocal models for HRSB and body bullying. A. Model 1: Auto-regressive effects and cross-sectional correlations; B. Model 2: Body bullying at T1 predicts HRSB at T2; C. Model 3: HRSB at T1 predict body bullying at T2; D. Model 4: Body bullying and HRSB have reciprocal effect. All models were adjusted by socioeconomic information and psychological well-being- omitted in the Fig 1.

**Fig 2:**
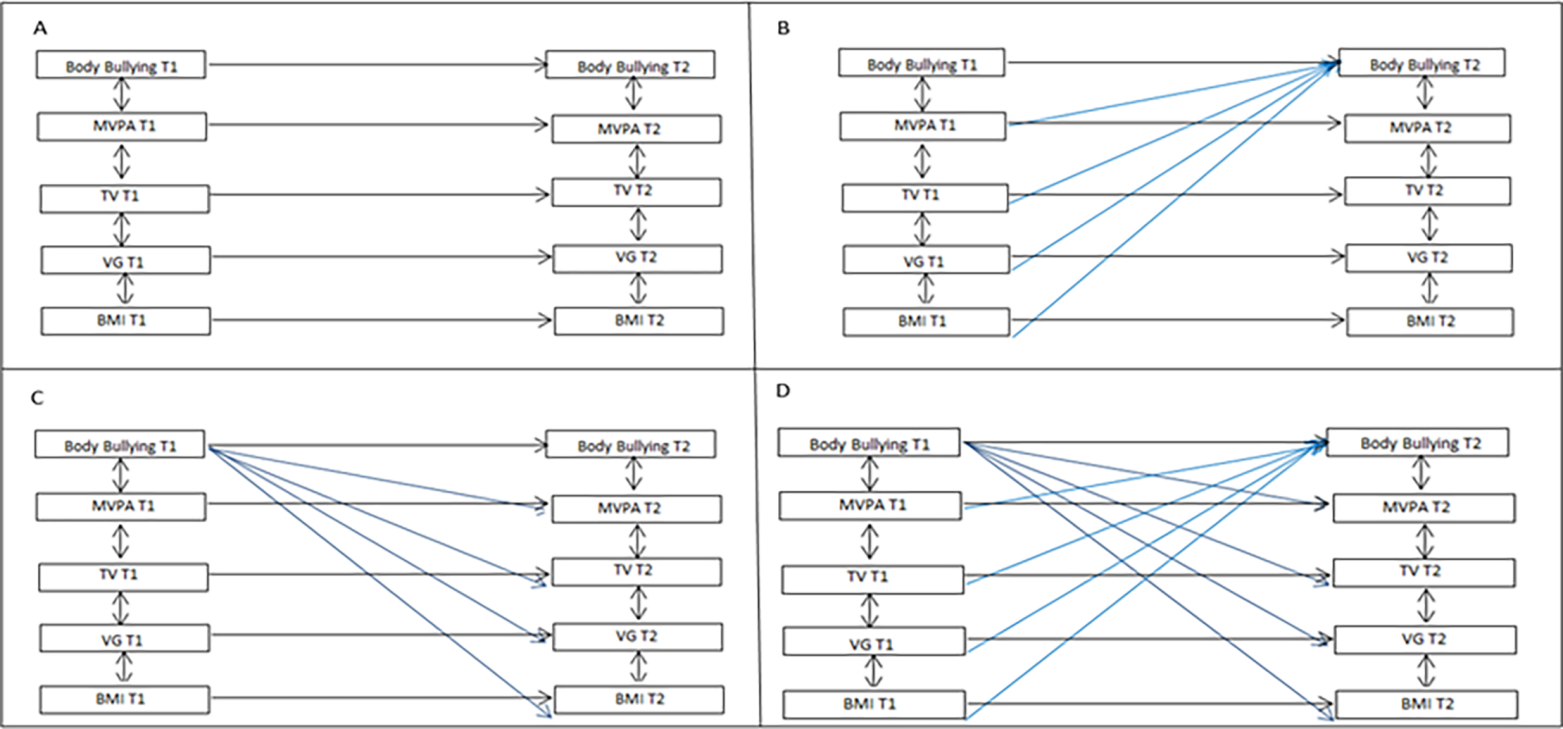
Structural equation modeling results and estimates (standardized) between HRBS and body bullying for boys/girls. *** p < .001, ** p < .01, * p < .05. Boys N = 436 and girls N = 374. All models were adjusted by socioeconomic information and psychological well-being- omitted in the Fig 2.

Based on the previous presented evidences of gender in this topic, we performed gender-specific autoregressive cross-lagged panel models to address simultaneously reciprocal influences on HRSB variables and body bullying by means of SEM, with maximum likelihood missing values estimation. Primarily, we tried to create one latent variable to represent all four HRSB (BMI, MVPA, TV, and VG) and then minimise measurement error of the models. However, the single HRSB items did not represent only one latent variable, so we performed the analyses using HRSB with single items (BMI, MVPA, TV, and VG). Therefore, we performed a baseline model of auto-regressive paths, which assess stability over time of each HRSB (T1 to T2) and body bullying (T1 to T2). This model also included correlations between all HRSB and body bullying at T1 and T2, respectively. Apart from the baseline model, we estimated three more models, including one or more cross-lagged effects and the confounder variables (type of school, asset ownership and psychological well-being- not shown in Figs 1 and 2). Fig 1 illustrates the models.

For each of the four models, indicators of model fit statistics was provided: the Standardised Root Mean Square Residual -RMSEA (satisfactory values below or close to 0.06), the Comparative Fit Index (CFI) and the Tucker-Lewis Index (TLI) (satisfactory values close to or above 0.95). Relative goodness of fit was provided through the comparison of The Akaike Information Criterion (AIC) and the Bayesian Information Criterion (BIC) that indicates the best fit those models with lower values. The chi-square difference tests were assessed to measure models that were nested hierarchies. We conducted all statistical analyses in Stata version 13.1 (StataCorp, Texas, USA).

## Results

The distribution of HRSB variables and body bullying can be seen in Table 1. Note that higher values consistently correspond to more bullying throughout the table. Gender differences were tested, showing that boys spend a significantly higher amount of time on MVPA and VG compared to girls at both T1 and T2. There were no statistically significant gender differences for the remaining items. With regard to changes over time (from T1 to T2), the results show that body bullying decreases significantly across the two time points for boys. For girls, a subtle increase of body bullying over time was presented, but this change was not statistically significant. Among both genders, there was a statistically significant increase in time spent on VG and an increase in BMI, as well as a decrease in time spent on MVPA.

**Table 1:** Distribution of the study variables. ELANA study, 2010 and 2012.

	N		Boys				N	Girls				Comparison boys-girls ^a^	
			Min.	Max.	Mean	St. dev.		Min.	Max.	Mean	St. dev.	Mean diff.	P value*
**Time 1**													
HRSB													
MVPA (minutes/day)	431		0.00	360.00	67.95	65.31	370	0.00	240.00	42.94	43.42	23.01	<0.001
TV (minutes/day)	432		0.00	540.00	195.03	158.08	371	0.00	540.00	200.37	172.57	-5.34	n.s.
VG (minutes/day)	435		0.00	540.00	142.11	145.97	371	0.00	540.00	104.42	133.11	37.69	<0.001
BMI (kg/m^2^)	427		12.80	39.80	20.42	4.31	365	12.60	38.90	20.10	4.30	0.32	n.s.
Body bullying ^b^	428		2.00	32.00	5.35	6.09	369	2.00	32.00	5.13	5.71	0.22	n.s.
**Time 2**													
HRSB													
MVPA (minutes/day)	288		0.00	231.40	41.26	41.20	259	0.00	300.00	29.12	37.79	12.14	<0.001
TV (minutes/day)	288		0.00	594.00	192.68	159.05	259	0.00	594.00	193.55	150.05	-0.87	n.s.
VG (minutes/day)	288		0.00	540.00	166.63	163.02	259	0.00	540.00	181.63	176.79	-15.00	<0.001
BMI (kg/m^2^)	283		13.60	37.20	21.45	4.23	254	14.36	38.70	21.51	4.31	-0.06	n.s.
Body bullying ^b^	282		2.00	32.00	4.35	4.88	254	2.00	32.00	5.31	5.88	-0.96	n.s.
**Comparison T2-T1** ^**c**^													
			Mean diff.		P value**			Mean diff.		P value**			
HRSB													
MVPA (minutes/day)			-26.60		<0.001			-13.82		<0.001			
TV (minutes/day)			-2.35		n.s.			-22.52		n.s.			
VG (minutes/day)			24.52		<0.001			43.68		<0.001			
BMI (kg/m^2^)			1.03		<0.001			0.01		<0.001			
Body bullying ^b^			-1.00		<0.05			0.17		n.s.			

Min: Minimum; max: Maximum; St. dev.: standard deviation; Mean diff.: Mean difference; n.s.: non significance; HRSB: Health-related state and behaviours; MVPA: Moderate and Vigorous Physical Activity; TV: Television; VG: Video game and computer; BMI: Body Mass Index

* Wilcoxon Test

** Wilcoxon Rank Compared Test.

^a^ A positive difference value reflects that boys have higher values compared to girls, whereas a negative difference value suggests the opposite.

^*b*^ Higher values in body bullying score indicate more bullying.

^c^ A positive difference value indicates higher values over time, whereas a negative difference value reflects the opposite.

As the first step of the SEM with maximum likelihood for missing values estimation, a baseline model was constructed with auto-regressive paths from BMI, MVPA, TV, and VG at T1 to T2 and from body bullying at T1 to T2. Moreover, correlations between HRSB variables and body bullying were added at T1 and T2, respectively.

The upper part of Table 2 shows the fit indices for boys whereas the lower part of the table gives the corresponding results for girls. For boys, all four models provide an acceptable fit to the data according to the values for RMSEA (0.016–0.033), CFI (0.971–0.995), and TLI (0.959–0.990). Model 4 has the lowest values for AIC and Model 1 for BIC. Moreover, the chi-square difference tests show that none of the Models 1–3 fit the data significantly better than Model 4. With regard to girls, the values for RMSEA (0.000–0.028), CFI (0.979–1.000), and TLI (0.969–1.004) indicate an acceptable fit for all four models. AIC is lowest for the forward causation model (Model 2) whereas the baseline model (Model 1) has the lowest BIC. According to the chi-square difference tests, Model 2 fits the data significantly better than Model 1, on condition that p<0.05 is the acceptable level. It should be noted here that Model 4 provides a significantly better fit for the data (p = 0.02) compared to Model 3 and Model 1 (p = 0.01), but not compared to Model 2.

**Table 2:** Goodness-of fit statistics for the tested models (n = 810). ELANA study, 2010 and 2012.

	Goodness-of-fit statistics			
	Model 1: Baseline^1^	Model 2: Forward causation^2^	Model 3: Reversed causation^3^	Model 4: Reciprocal^4^
**Boys**				
RMSEA	0.033	0.026	0.027	0.016
CFI	0.971	0.984	0.983	0.995
TLI	0.959	0.974	0.973	0.990
AIC	38841.572	38838.476	38838.897	38836.225
BIC	39122.930	39136.144	39136.565	39150.204
*χ*^2^	51.41	40.31	40.74	30.06
*Df*	35	31	31	27
*P*	0.036	0.122	0.113	0.311
Chi-square difference test				
Comparison with:	-	Model 1	Model 1	Model 1/Model 2/Model 3
Change in *χ*^2^	-	11.1	10.67	21.35/10.25/10.68
Change in *df*	-	4	4	8/4/4
*P*	-	0.02	0.03	<0.05/0.03/0.03
**Girls**				
RMSEA	0.028	0.014	0.023	0.000
CFI	0.979	0.995	0.987	1.000
TLI	0.969	0.992	0.979	1.004
AIC	33623.279	33619.319	33623.362	33619.949
BIC	33894.052	33905.790	33909.833	33922.116
*χ*^2^	45.29	33.33	37.38	25.96
*Df*	35	31	31	27
*P*	0.114	0.354	0.199	0.520
Chi-square difference test				
Comparison with:	-	Model 1	Model 1	Model 1/Model 2/Model 3
Change in *χ*^2^	-	11.96	7.91	19.33/7.37/11.42
Change in *df*	-	4	4	4/4/8
*P*	-	0.01	0.09	0.01/0.11/0.02

^1^ Only auto-regressive effects and cross-sectional correlations.

^2^ Body bullying at T1 predicts health- related state and behaviours at T2.

^3^ Health- related state and behaviours at T1 predict body bullying at T2.

^4^ Body bullying and health- related state and behaviours have reciprocal effect.

Based on the model fit statistics, it was decided to proceed with Model 4 for boys and Model 2 for girls. The results from structural equation modelling are shown in Fig 2. For clarity, only the significant paths are shown, but all variable correlations were included. Moreover, the error terms and the confounding variables–psychological well-being, type of school, and asset ownership–have also been omitted from the Fig 2.

With regard to the auto-regressive paths, the coefficients for HRSB were 0.008 (p>0.05) for MVPA, 0.28 (p<0.001) for TV, 0.35 (p<0.001) for VG and 0.85 for BMI among boys; for girls 0.09 (p>0.05) for MVPA, 0.07 (p>0.05) for TV, 0.24 (p<0.001) for VG and 0.86 for BMI (p<0.001). For body bullying, the stability coefficients were 0.41 (p<0.001) and 0.44 (p<0.001) for boys and girls, respectively. The results showed a positive correlation between BMI and body bullying (0.32, p<0.001) for boys, and between TV and VG for boys (0.24, p<0.001) and girls (0.30, p<0.001 and 0.30, p<0.001), at T1. The strength of the correlations diminished over time, but remained significant at T2 (boys: 0.18, p<0.05 and 0.16, p<0.01; girls: 0.21, p<0.01 and 0.22, p<0.01). With regard to the cross-lagged effects, the results suggest that body bullying at T1 predicts an increase in girls’ time spent on VG from T1 to T2 (0.16, p<0.001). Finally, for boys, the results showed a significant correlation between BMI at T1 and body bullying at T2 (0.12, p<0.01) and between the body bullying at T1 and VG at T2 (0.14 p<0.01).

## Discussion

This study examined changes in BMI, MVPA, TV, and VG and body bullying as well as the correlations between measures. Using SEM for longitudinal data, this study investigated the causal directions and reverse causality of four HRSB (BMI, MVPA, TV, and VG) and being body bullied.

During follow-up, an increase in BMI was observed for boys and girls, and this result is consistent with the literature [36, 37]. Despite the lack of statistically significant differences between genders, obesogenic factors could influence weight gain in adolescence and should be looked at carefully [38, 39]. Due to the influence of sexual maturation, the increase of body fat mass is faster in girls than in boys, whose increase is surpassed by the gain of body fat free mass [36, 40]. A significant decrease in time spent on MVPA was observed in both genders over time. The first prospective data from low/middle-income countries realised in Brazil concerning PA changes in adolescents found that boys increased their leisure-time PA level over the four years, whereas a decrease was observed among girls [41].

Regarding screen time, a significant increase of VG time and a non-significant decrease in TV was observed for boys and girls over time. The reduction in TV watching and increase in VG have been observed in 30 developed countries, as Bucksch et al. (2016) [42] have shown in a recent international trends study among boys and girls, as well as in another study in the southern region of Brazil [22]. In Brazil, the first few years of the 2000s were characterised by a reduction in the social gap between the rich and the poor through a combination of unemployment reduction, progressive increases in the minimum wage and the expansion of cash transfer programs, reflected in an increase of TV and computer acquisitions in Brazilian households [21–23].

At T2, boys had a significant decrease in body bullying scores, while girls had a non-significant increase. Longitudinal samples from Project EAT-II (Eating Among Teens–II), Minnesota- US, reported a higher prevalence of weight teasing among girls in both waves analysed (1998/99 and 2003/04) [8].

Based on the results from SEM (taking into account the autoregressive effects) BMI and body bullying, the amount of TV and VG were found to be correlated at each separate time point for girls and boys. The strength of the correlations diminished over time, but remained significant at T2. The correlation between BMI and body bullying that was stronger for boys at T1 became stronger for girls at T2.

Despite some evidence in the literature having shown that bullying in middle school is more severe, frequent, and upsetting for overweight youth [12], Haines et al. (2008) [43] demonstrated, in longitudinal trends from Project EAT (Eating Among Teens–II) in Minnesota-US, that weight-related teasing decreased among female and male overweight adolescents as they transitioned from early adolescence to mid-adolescence [43].

For instance, most students have their first experience of sports practice at the beginning of adolescence. These experiences generally provide greater exposure of the body, and consequently, adolescents could be more likely to be evaluated by their peers on the excess of body fat, which is frequently recognised by practitioners as having a negative influence on performance. In this context, body bullying might be especially harmful among boys since they tend to participate in more PA in this age group than girls [43]. On the other hand, girls in the middle of adolescence may have aesthetic and body image concerns as a focus [12, 26]. The mass media is depicting increasingly leaner women as the societal ideal, and research reveals that internalising these images increases body image disturbance, negative mood, and eating disorder symptoms [44, 45]. Thus, boys’ body image experiences mirror those of girls but are typically less prevalent, and when present, less severe [46].

The goodness-of-fit statistics showed the reciprocal model with cross-lagged effects provided the best fit for boys. Thus, the association between body bullying and HRSB should be interpreted as a bi-directional relationship, considering the cross-lagged effect between BMI (T1) and body bullying (T2)/ body bullying (T1) and VG (T2). The forward causation model was identified as the most suitable for the female sample–a conclusion that stands on shaky grounds as the model-fit comparison between the reciprocal model with cross-lagged effects and forward causation model showed a p-value in favour of the latter one. Nonetheless, the forward causation model suggested a weak cross-lagged effect of body bullying at T1 on VG at T2.

This could be interpreted as boys and girls that are bullied being more likely to resort to VG, but not TV. Research concerning motivation for playing video games in adolescents from the US, aged between 12 and 14 years old, poses that among the group that cited ‘make friends’ as a motivation for playing video games, more adolescents were likely to be victims of bullying and to report being excluded by their peers; thus, they may put a higher value on connecting with peers through video games [47]. Meta-analytical research regarding video games and effect on social outcomes indicates that video game exposure causally affects social outcomes and that there are both short- and long-term effects [48]. Meanwhile, the literature has also shown the importance of TV time and bullying in adolescents. Rech et al., (2013) [4] found that South Brazilian schoolchildren from both genders with more than three hours a day spent on TV, computer and VG were 55% more likely to be victims of bullying (RR = 1.55; CI = 1.01–2.36). Another study conducted in eight countries (Canada, Estonia, Israel, Latvia, Macedonia, Poland, Portugal, and the United States) also showed an association between the number of hours watching TV and bullying [19].

Regarding the autoregressive effect between the amount of TV and VG, even if both activities reflect sedentary behaviour and are possibly correlated, the decrease in TV watching appears to be compensated by the increase in computer/video game use in adolescents [22]. Despite the fact that the undesired psychological processes associated with exposure to TV and electronic games are similar, those who play games may experience worse symptoms due to active user engagement, identification with characters and repeated rehearsal and reinforcement. Gaming’s interactive and absorbing qualities may act as a substitute for interpersonal relationships and increase social isolation. Such isolation may provoke anxiety and depression, or, if coupled with reduced empathy may decrease prosocial behaviour [49].

Few cross-sectional studies have demonstrated that victimised overweight youth are less likely to be physically active [26, 50, 51]. A study from a German secondary school reported more frequent weight teasing was associated with decreased PA in boys; it was also observed that overweight participants reported more frequent weight teasing experiences and less self-efficacy than participants of normal weight, but there was no difference in PA [17]. Slater and Tiggemann (2011) [25] concluded that teasing and body image concerns may contribute to adolescent girls’ reduced rates of participation in sports and other PAs among adolescents from South Australia. However, almost all of these studies were developed with cross-sectional data, finding punctual associations. The fact that the proposed model presented in this study considers the complexity of the evaluated scenery in which the joint effect of all variables was included, could be one explanation for the absence of effects concerning PA. For example, omitting an important cross-lagged effect in an estimated model can bias the other parameter estimates. Hence, when a robust model was used to compare an isolated association, more approximate effect estimates of the complexity of the studied problem can be gauged [52].

### Limitations, strengths and implications

There are some drawbacks that should be considered. Due to the follow-up sampling procedure, missing cases are relatively high from T1 to T2. As we were aware of the constraints regarding the imputation process, it seems necessary to lead with this methodological issue. We based our results on self-reported information of PA and screen time, with non-additional information from the parents. In spite of this potential limitations may still include the adolescent’s ability to remember, interpret, and quantify time spent on these activities [53, 54], the possibility of information bias would not affect the outcome, since it would tend to be random, not differential for those with or without the outcome. Moreover, it is important to consider that information from parents could entail problems such as over or underreporting of children’s negative behaviours and be unaware about the actual time spend on screen activities.

Related to the measure of body bullying, the absence of assessment of a power imbalance between the perpetrator and the victim could be also cited as a limitation. The causal lag of 2 years identified effects of being body bullied during the transition from early to middle adolescence. However, a long lag time may have shown larger effects, covering the end of adolescence and the transition to early adulthood.

Despite of the limitations, a strength of this study is the unique data sample that evaluated body-bullying data at two time points in a middle-income country, rendering it possible to account for cross-lagged and autoregressive pathways. Demonstrating the directionality of the relationships becomes important from a theoretical, practical, and methodological perspective. The SEM analyses freely estimates regression coefficients without imposing equality constraints; thus, potential changes in the strength of causal relations over time can be detected taking into account the synergetic actuation of the HRSB in this context.

Thus, understanding how these social determinants of health act together to create a very complex context can have important implications for public health and can be used to assist in the development of health prevention initiatives and in guiding the work of school managers. Interventions that are properly created to change multiple behaviours can be cost-effective and maximise the reach, which is important in cases where the availability of funds are reduced and/or socioeconomic conditions are not favourable. There is relevance in promoting universal approaches to discuss social and health consequences of body bullying and HRSB in school and communities with all children and adolescents. Although, target actions for more susceptible groups and specific techniques of coping for boys and girls could be, also more efficient.

## Conclusion

This is the first time that a study using SEM has highlighted the complex interplay between body bullying and HRSB (BMI, MVPA, TV and VG), as well as the importance of acknowledging gender differences in these associations in adolescents in a middle-income country context. Being overweight seems to influence boys to be bullied and also those bullied to spend more time on VG, showing a bidirectional association between body bullying and HRSB for boys. Among girls, we found that body bullying had an effect on VG time, but no effect was detected in the opposite direction. PA does not seem to be affected by or affect body bullying in either gender. Future studies in this context using direct measurements of PA and sedentary time should be considered. Furthermore, investigations with more evaluations over time, covering the whole period of adolescence, as well as robust methods such as SEM, which deal with a complex reality could provide important new evidence in this field of research.

## Abbreviations

AIC: Akaike Information Criterion
BIC: Bayesian Information Criterion
CATS: Child-Adolescent Teasing Scale
CI: Confidence Interval
CFI: Comparative Fit Index
BMI: Body Mass Index
ELANA: The Adolescent Nutritional Assessment Longitudinal Study
HRSB: Health-Related State and Behaviours
IPAQ: International Physical Activity Questionnaire
MVPA: Moderate and Vigorous Physical Activity
PA: Physical Activity
RMSEA: Standardised Root Mean Square Residual
SEM: Structural Equation Models
T1: Time 1
T2: Time 2
TLI: Tucker-Lewis Index
TV: Television
VG: Video game and computer
